# Domestic violence assault during the first year of the COVID-19 pandemic: a longitudinal community study

**DOI:** 10.1186/s12889-023-15560-8

**Published:** 2023-04-20

**Authors:** Yasmin B. Kofman, Cassidy C. D. Weiss, Ilona S. Yim

**Affiliations:** 1 Department of Psychology, University of California, Los Angeles, Los Angeles, USA; 2 Donna Ford Attallah College of Educational Studies, Chapman University, Orange, USA; 3 Department of Psychological Science, University of California, Irvine, USA

**Keywords:** Domestic violence, COVID-19, Calls for service, Pandemic, Assault, Community

## Abstract

**Background:**

The consequences of the COVID-19 pandemic have been far-reaching, disproportionately impacting vulnerable populations. Of particular concern is the impact on individuals experiencing domestic violence (DV), an urgent public health issue. There have been numerous reports of pandemic-related surges in DV, and it has been speculated that prolonged periods of state-mandated isolation may be the source of these surges. The current study utilized publicly available records to examine fluctuations in DV coinciding with COVID-19 lockdown restrictions in a diverse metropolitan county.

**Methods:**

Data were extracted from local police blotters and mapping engines in Orange County, California (United States), documenting police-reported DV assault. All incidents were coded for time to examine the time course of DV among other types of assault, allowing for a longitudinal view of incidents over a 66-week window. Changepoint analyses were used to determine whether and when DV assaults changed when mapped with coinciding tightening or loosening of restrictions county-wide. Piecewise regression analyses evaluated whether any detected fluctuations were statistically meaningful.

**Results:**

In Santa Ana, rates saw a small but significant spike in the week following the first major lockdown in March 2020 (*b* = .04, *SE* = .02,* t* = 2.37, *p* = .01), remaining stable at this higher level thereafter (*b* = -.003, *SE* = .003, *t* = -1.29, *p* = .20). In Anaheim, no meaningful change in DV assault rates was observed at any time interval.

**Conclusion:**

Results suggest that surges in DV vary between communities and that systemic issues may set the stage for the surge of an already endemic problem.

The COVID-19 pandemic has had far-reaching consequences, uniquely impacting domestic violence (DV) victims and survivors. DV is often used interchangeably with intimate partner violence, a pattern of psychological, emotional, physical, or sexual violence or abuse committed by a current or former intimate partner [1]; however, it should be noted that DV encompasses family violence more broadly, with slightly varying definitions across different jurisdictions. Considered by the Centers for Disease Control (CDC) an urgent public health issue, DV has reached disturbingly high levels in the United States (U.S.), a surge noted immediately by police, news, and technical reports after the first unprecedented nationwide lockdown in March 2020 [2].

Increases in DV during a collective crisis are not new. Surges have been documented after disasters and anthropogenic events, and although guidelines have been suggested for implementation at various levels of government [3], they are rarely implemented comprehensively. The current pandemic has produced a unique set of challenges for DV victims and survivors to grapple with [2]. Most notably, there have been periods of prolonged isolation due to sweeping stay-at-home mandates, economic downturn and displacement, and uncertainty, which have kept victims trapped and under the coercive control of abusers. One particularly alarming facet of the current circumstance is that victims have been closed off to avenues typically used to alert others to an abusive situation. For example, many non-urgent and non-essential medical procedures and appointments were initially postponed or canceled, eliminating a vital point of contact for abuse to be disclosed or detected by medical professionals [4]. Even throughout re-openings, many service providers maintained remote contact, opting for telehealth to keep medical staff and patients safe and preempt any potential COVID-19 spikes and restrictions. Additionally, being in close and constant proximity to violent partners, either as a result of a lockdown, remote work, or loss of employment or furlough, potentially creates a dangerous environment in which victims cannot safely access DV-related resources like websites, textlines, and hotlines. Therefore, a victim’s only or last resort might be to call the police (or have a bystander make the emergency call) once violence has escalated to the point where there is immediate danger of physical harm, injury, or death. Still, research shows domestic violence and other forms of gender-based violence are grossly underreported, even in an emergency [5].

While awaiting federal and state response to both the pandemic and the current DV crisis, it is important for researchers to bring awareness to the immediate and heightened levels of danger victims find themselves in, and in this way, mobilize support for communities in need. The current study aims to utilize publicly available police records to examine whether there was a rise in DV that necessitated an emergency call (i.e., reported and documented by police as an assault), potentially due to events surrounding COVID-19 lockdown restrictions, in a diverse metropolitan county. Because most available reports—though anecdotal—suggest that DV generally has been on the rise since lockdowns began in March 2020, it was hypothesized that there would also be an increase in DV assaults after the first local lockdown. Fluctuations in DV have not yet been reported; thus, an exploratory aim was to examine whether levels of DV assault fluctuated in accordance with tightening and loosening of restrictions.

## Method

### County description and timeline

Orange County is a metropolitan region in Southern California. With about 3 million residents, it is the third-largest county in the state after San Diego and neighboring Los Angeles County and the sixth-most populous county in the United States [6]. Traditionally, Orange County has a reputation for being an affluent, conservative-leaning, mostly demographically homogeneous county. However, over the past several decades, the county has undergone significant demographic shifts [7]. For example, the most recent U.S. Census Bureau Report (2019) indicates that in terms of ethnic and racial demographics, Orange County is about 40% White (non-Hispanic), 34% Hispanic/Latino, and about 22% Asian. The most striking differences in county demographics are seen between North/Central Orange County (e.g., Anaheim, Santa Ana, Westminster) and South Orange County (e.g., Newport Beach, Laguna Beach, San Clemente). Half of all Latinos in Orange County live in Santa Ana and Anaheim [7], which are two of the most densely populated cities in the county (and in the country), with among the lowest income per capita [6]. In contrast, South Orange County cities have the highest income per capita in the county [6]. In terms of violent crime, levels have remained relatively stable from 2006 to 2019, and Orange County is in the best 50% of counties in the U.S. in terms of violent crime distribution [8].

#### Pandemic timeline of events in Orange County (January 2020 – March 2021)

**Fig. 1:**
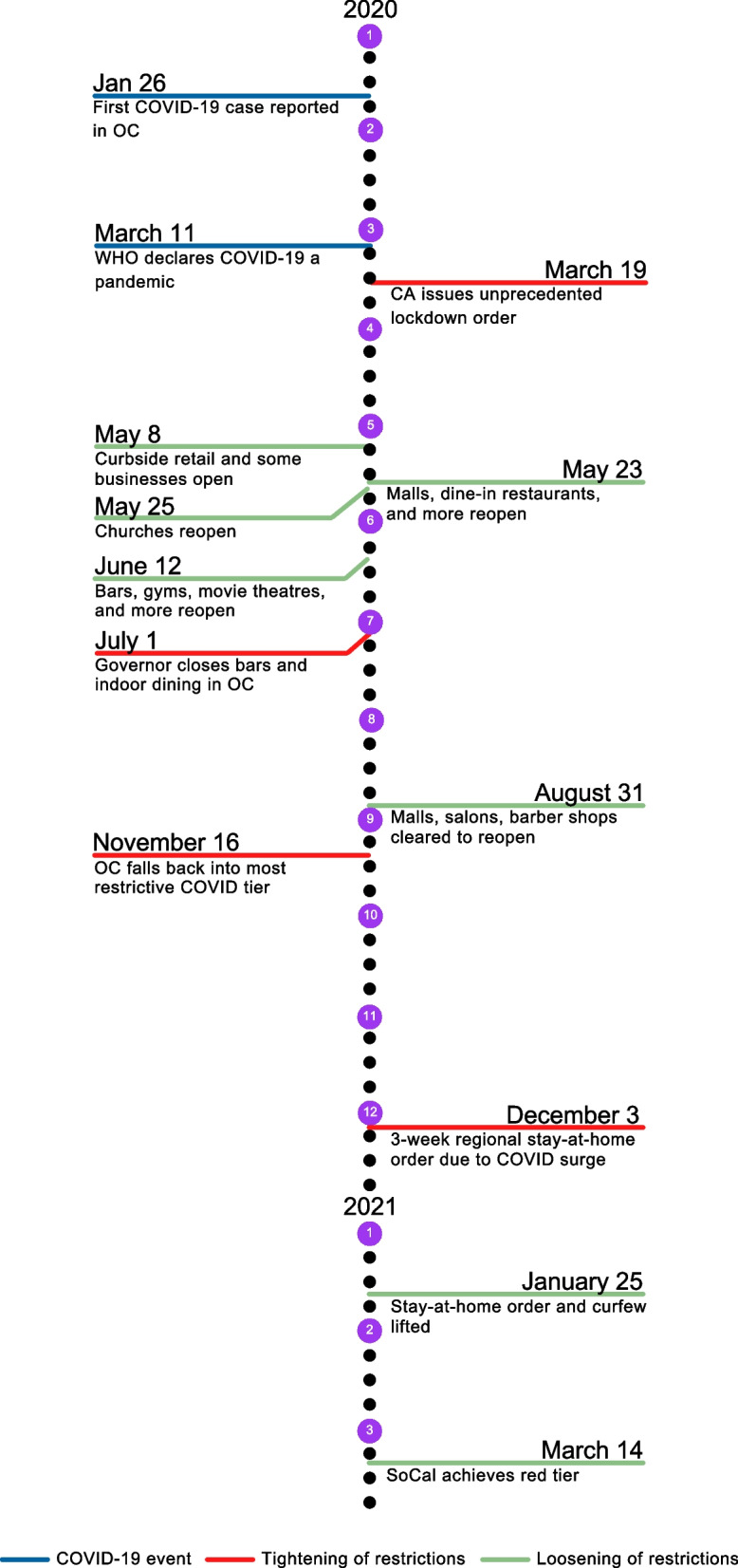
Southern California county-wide restrictions from January1st, 2020 (11 weeks prior to first lockdown) to March 31^st^, 2021 (54 weeks after the first lockdown). CA = California; OC = Orange County; SoCal = Southern California

As several California counties began declaring public health emergencies in February, starting in March, the state imposed some of the strictest lockdowns in the country in an effort to flatten the coronavirus curve. Several notable lockdown events occurred statewide and in Orange County. On March 13^th^, all schools were closed, followed by a ban on gatherings and in-person dining. Finally, on March 19^th^, all but essential services were shut down—a statewide order. Restrictions did not begin to loosen until the end of May when some non-essential services (dine-in restaurants, malls) were allowed to reopen. On June 12^th^, most other non-essential businesses (gyms, personal care services, theaters) were allowed to reopen again. This reopening was short-lived, and on July 1^st^, all Orange County indoor dining and family entertainment was closed, followed by a shut-down of all indoor operations for non-essential personal care services (e.g., salons), gyms, places of worship, indoor malls, and offices on July 13^th^. A week later, salons and barbershops were allowed to resume outdoor services only (if they had the capacity to do so). At the end of August, Orange County was moved off the coronavirus “watchlist,” a monitoring threshold based on key metrics like hospitalizations and positive infection rate. Still, most non-essential businesses would remain closed until the beginning of September, after California’s watchlist monitoring system was replaced with a tier-based, color-coded reopening plan based on coronavirus spread in each county (Purple [Widespread], Red [Substantial], Orange [Moderate], Yellow [Minimal], [9]). This would allow a limited number of sectors, like indoor dining, to reopen at reduced capacity. In December 2020, California enacted the strictest lockdown since March 2020 (in an official statement, the Orange County Sheriff said he would not enforce this lockdown) following record-breaking spikes in COVID-19, which was lifted approximately a month later, at the end of January 2021. Finally, in March 2021, Orange County moved into the less restrictive Red Tier, once again allowing re-openings for various sectors. Of note, Disneyland Resort, Orange County’s largest employer located in the city of Anaheim (Woods Center for Economic Analysis and Forecasting, 2019), remained shut down from March 2020 to April 2021 and did not reopen at any point during that time, affecting over 20,000 local employees. A general timeline of tightened and eased restrictions in Orange County is shown in Fig. 1 (see Procter [10] for a comprehensive timeline of events in Orange County).

### Data extraction and cleaning

Data were manually extracted from local police blotters and mapping engines, which continuously extract calls for service and reports from participating law enforcement agencies’ records systems. Data through these sources are verified for accuracy by the participating sites, and location information is generalized by neighborhood block to ensure confidentiality. Fifteen broad categories of crimes are captured: Arson, Assault, Burglary, Disturbing the Peace, Drugs/Alcohol Violations, DUI, Fraud, Homicide, Moto Vehicle Theft, Robbery, Sex Crimes, Theft/Larceny, Vandalism, Vehicle Break-In/Theft, Weapons, Sex Offender, Sexual Predator. For the purpose of this study, only incidents categorized under “Assault,” which is broadly defined as an attack on a person to commit injury (California Penal Code 240), were extracted from January 1^st^, 2020 through March 31^st^, 2021. Data from May 1^st^ through June 31^st^ were unavailable for both agencies at the time of extraction and could not be retroactively accessed because of the 180-day limit on data availability. Likewise, data from October 7^th^, 2020 through December 17^th^, 2020 were unavailable for Santa Ana. Online crime mappers are dependent upon participating police agencies to reflect corresponding data. It is possible that agencies experienced technical difficulties that interrupted data loading. We attempted to contact the participating agencies directly but were not able to obtain the missing data.

#### Assault incidents

**Fig. 2:**
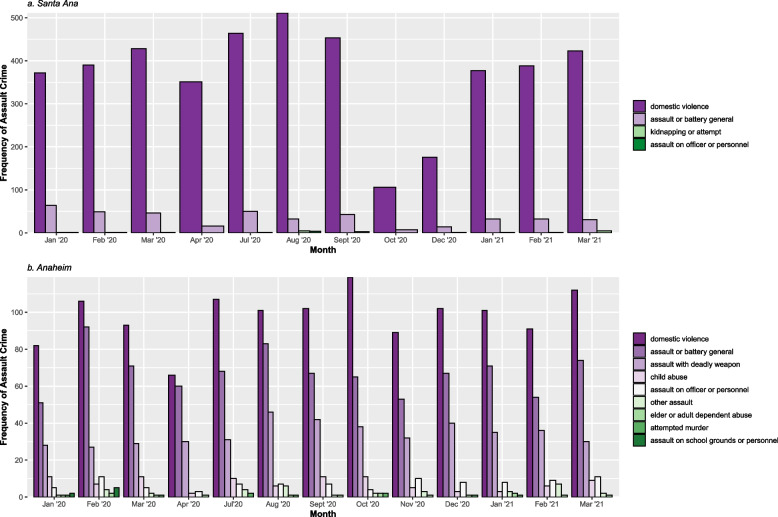
Types of assault crimes per month as for Santa Ana (**a**) and Anaheim (**b**). Note that the y-axis is not on the same scale

Data were prepared to examine rates of DV assault versus other types of assault. To consolidate domestic violence assault incidents, only incidents that explicitly indicated domestic violence were recoded as such (i.e., Battery-Spouse, Beat Spouse, Domestic Battery, Domestic Violence, Domestic Violence Battery). Only two out of the 20 participating Orange County police agencies, Anaheim and Santa Ana, provided these types of DV-specific data and thus were the only two agencies included in all subsequent analyses. All other assault incidents, a total of 31 unique penal codes, and an additional three types of crime that did not have any corresponding penal code (i.e., Assault and Battery, Assault on Officers, Kidnap and Attempts) were consolidated and recoded into ten broad categories based on the penal code description (see Table 1). In total, 7,488 assault incidents, including DV, were extracted in Santa Ana and Anaheim. After cleaning and recoding these data, and upon visualizing the monthly distribution of assault crimes by agency (see Fig. 2a and b), it was noted that only the Anaheim Police Department provided specific penal codes and for a variety of assault crimes. Santa Ana Police Department, on the other hand, did not provide penal codes and only provided the broad descriptive categories of Domestic Violence, Assault and Battery, Assault on Officers, and Kidnap Attempts. Because of this discrepancy in reporting, all subsequent analyses were done separately for each agency. Given the relatively low levels of specific assault types (e.g., kidnapping, elder abuse, assault on school grounds), assault incidents were dichotomized, coding all DV specific incidents as 1 and all other assault types as 0 for consistency and ease of interpretation (see Fig. 2 for frequency of DV versus other assault incidents by agency).

**Table 1:** Recoding of penal codes and/or descriptions to broad assault crime categories

Assault crime category	Penal code and/or description
Domestic violence assault	243(E)1 BATTERY- SPOUSE/ETC 273.5(A) BEAT SPOUSE/CO- HABITA **DOMESTIC VIOLENCE**
Child abuse/cruelty	273A(A)CRUELTY TO CHILD- INJURY 273A(A)WILLFUL CRUELTY CHILD 273A(B)CHILD ENDNGRMNT- PHYSCL 273D(A)PC INFLICT INJ ON CHILD
Assault with a deadly weapon	245(A)(1)ADW/NO FIREARM/CIVIL 245(A)(2) ADW/FIREARM/CIVILIA 245(A)(3) ADW ASSLT WEAPON 245(A)(4) ASSLT/FORCE/GBI 245(B) PC ADW SEMIAUTO FIREAR
Elder/dependent adult abuse/cruelty	243.25PC BATTERY AGAINST ELDER 368(B) CRUELTY TO DEPEND ADULT 368(C) CAUSE PAIN/INJ TO ELDER
Assault/battery on officer/emergency personnel	148.10(A)OBST/RESIST/FORCE/SBI 241(B) ASLT ON PO/EMERG PRSNL 241(C) ASSLT ON PO/FRFGHTR/ETC 243(B) PC BATTERY-OFFICER/ETC 243(C)(1)BATT EMERG PERSON-INJ 243(C)2 PC BATTERY-POLICE OFCR 245(C) ADW/NO FIREARM/OFFICER 245(D)(2)ADW/SEMIAUTO/OFFICER 247.5 DSCHRG LASR-POLICE ARCRF 69 PC RESIST EXEC OFFICER 69(A) PC RESIST EXEC OFFICER **ASSAULT ON OFFICERS**
Assault on school grounds or personnel	241.2A ASSLT ON SCHOOL GROUND 241.6 ASSAULT ON SCHOOL EMPL 243.2(A)(1)BATT/SCHL GRNDS-INJ
Attempted murder	664/187(A) ATT HOMI/AGG ASSLT
Kidnap or attempt	**KIDNAP AND ATTEMPTS**
Assault/battery (general)	203 PC MAYHEM 240 PC ASSAULT/SIMPLE 242 PC BATTERY/SIMPLE 240 PC ASSAULT/SIMPLE 243(D) PC BATTERY SERIOUS INJ **ASSAULT AND BATTERY**
Other assault	244 PC ASSAULT W/ CAUST CHEMIC 246 PC SHOOT OCCUP DWELL/VEH 23110(B) VC THROW AT VEH W/INT

Assault crimes for Santa Ana bolded (penal codes not provided by police agency or mapping engine)

#### Assault incidents pre- and post-first lockdown

All incidents were coded for time to examine the time course of DV among other types of assault. The timestamp of the assault incident, which can reflect the occurred time or reported time, was used to group incidents by week from the first lockdown (from -11 weeks to 54 weeks, relative to March 19^th^, 2020), allowing for a longitudinal view of incidents. Using the dichotomized assault incident variable (DV = 1, Assault [general] = 0), a proportion score was created for each assault incident to reflect the proportion of DV assault incidents to all other assault incidents for each week.

## Data analysis

All data were statistically analyzed in RStudio [11] and visualized using *ggplot* [12] using procedures outlined by Jones and Silver [13]. A general additive model smoothing function was also used for descriptive plots to show nonlinear line-of-best-fit. Trajectories of DV assaults before and after the first lockdown were evaluated over a 66-week window. Weeks with missing data (Weeks 5–13 both cities; Weeks 29–37 Santa Ana) were treated as missing in all analyses.

### 66-week window

The proportion of DV assault incidents was calculated for each of the roughly 11 weeks preceding the first lockdown to 54 weeks after the first week of lockdown. As delineated in Jones and Silver [13], a changepoint analysis was run using the *changepoint* package [14] to determine whether and when DV assaults increased, decreased, or stayed the same over this 66-week window. While a change was anticipated post-first lockdown, this analysis also served to explore whether there were other meaningful inflection points across this period, presumably coinciding with the tightening or loosening of restrictions. When spikes in DV assault were observed, a piecewise regression analysis was then utilized. A “knot” was placed at the week(s) that changepoint analyses indicated as the interval at which DV assaults stabilized at a higher level.

## Results

**Fig. 3:**
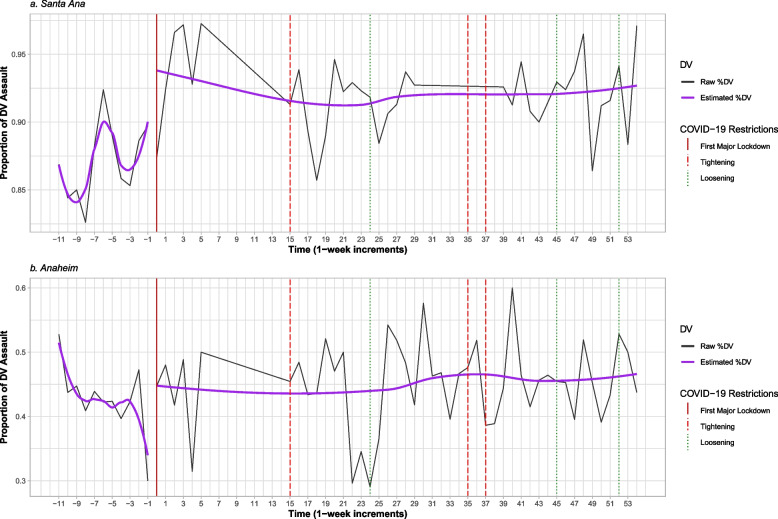
Domestic violence assaults for Santa Ana (**a**; *n*_DV_ = 4,439, *n*_assaults_ = 4,881) and Anaheim (**b**; *n*_DV_ = 1,271, *n*_assaults_ = 1,576) 11 weeks before and 54 weeks after the first week of lockdown. Solid vertical line depicts week of first major lockdown

Weekly proportions of DV assault incidents across all available data are shown separately for Santa Ana (see Fig. 3a) and Anaheim (see Fig. 3b). In both cities, rates of DV assault were substantial pre-lockdown; however, proportions of DV relative to other types of assault were strikingly high in Santa Ana, with 85% to 90% of assaults being DV-related before the first major lockdown. A couple of peaks in DV were observed at rates of about 95% around the time after the first lockdown and in the weeks following a strict 3-week lockdown in December, with the gradual easing of restrictions shortly thereafter. In Anaheim, proportions of DV relative to other assaults before the first major lockdown hovered between 30 and 50% and appeared to stay between 40 and 50% on average in the weeks thereafter.

**Fig. 4:**
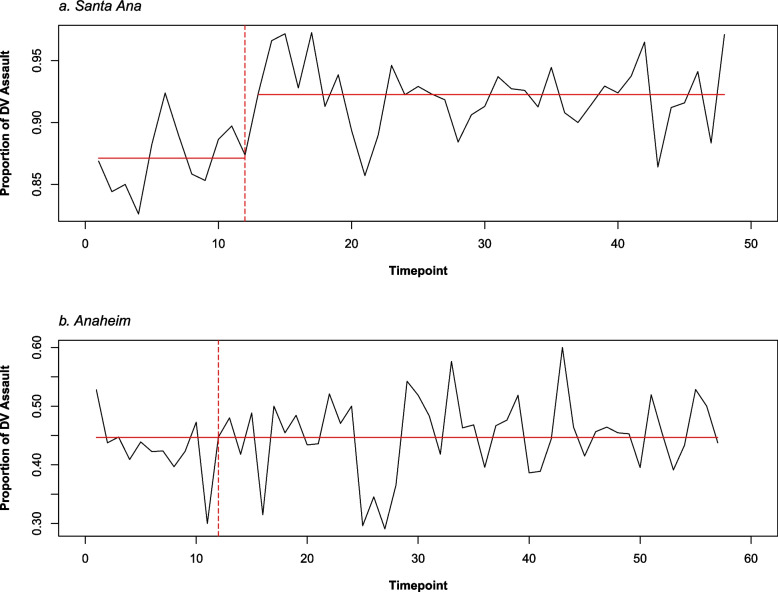
Changepoint analyses for Santa Ana (**a**) and Anaheim (**b**), in which Time 12 (vertical dashed line) is the first week of lockdown in Orange County. Note that Santa Ana has fewer number of timepoints given missing data

Changepoint analyses in Santa Ana indicated that DV assaults remained stable before the first lockdown and spiked in the week following the initial lockdown week, remaining stable at this higher level in the following weeks (see Fig. 4a). Consistent with the hypothesis that DV assault would increase post-lockdown and confirming what is seen descriptively in Fig. 3a, DV assault incidents in Santa Ana increased by 4% in the week following the initial lockdown week (*b* = *0.04, SE* = *0.02, t* = *2.37, p* = *0.01*), a small but significant surge. The slope of the lines before and after the first week of lockdown was not significantly different, confirming that levels of DV assaults remained relatively stable after the initial spike in DV (*b* = *-0.003, SE* = *0.003, t* = *-1.29, p* = *0.20*). Changepoint analyses for Anaheim indicated no meaningful change at any time interval, and DV assaults remained relatively stable (see Fig. 4b); thus, no further analyses were conducted.

## Discussion

This study used publicly available police data to examine whether DV assault, as indicated by an emergency call or documented police report, increased after the first major lockdown in Orange County, a major metropolitan area in Southern California. Results showed that there can be variation within a region, and only Santa Ana saw a surge in the proportion of DV assault after the first week of lockdown. Importantly, this surge was sustained across the 54 weeks for which we have follow-up data. Furthermore, despite several lockdown-related events (i.e., loosening and tightening of restrictions) being documented across the time that data were extracted after the initial lockdown, levels of DV assault did not fluctuate. Notably (and perhaps shockingly), the proportion of DV assault to other types of assaults was already extremely high in Santa Ana (around 80%-90%) and mainly stayed above 90% after the initial lockdown surge. This indicates that almost all calls for service for assault were DV-related.

There are several possible reasons for the small but significant surge seen in Santa Ana. First, disaster-related stress has been shown to precede DV perpetration [15]. When coupled with dwindling resources in an already under-resourced community, these factors may synergize to produce a surge in DV. As mentioned, Santa Ana is the most densely populated city in Orange County, with the lowest income per capita. While still considered predominantly low-income, Anaheim — home to Orange County’s largest employer, The Walt Disney Company — has about half the population density with a per capita income of about 1.4 times that of Santa Ana. This, along with a high level of income inequality and severe housing problems, like overcrowding and high housing costs that are documented in Santa Ana [16], are significant, systemic-level risk factors for DV [17, 18]. A palpable mobilization of resources to mitigate these issues was not felt in most areas until after the American Rescue Plan Act was passed in March 2021 [19]. This bill included critical funding for services and programs aiding DV victims and DV survivors, such as housing vouchers. This type of support is especially critical in areas like Orange County, home to many immigrant and mixed-status families, where undocumented survivors face additional barriers to help-seeking. Still, these supports must remain in place in the long-term, not just in the acute phase of COVID-19, as results here signal that economic and housing security play a significant role in survivors’ safety. Second, it is possible that, given the community context, the factors mentioned earlier are central to whether or not DV rates fluctuate in the face of a crisis, as opposed to various lockdowns and openings. In Santa Ana and Anaheim, most residents work essential jobs in manufacturing and accommodation and food service [20], potentially making the impact of various restrictions moot.

The data here add to a small but growing body of empirical literature documenting an overall increase in DV as collateral of well-intentioned and important policies implemented to reduce the coronavirus spread (see Piquero [21] for a meta-analysis of 12 U.S. studies). Particularly relevant are results from two studies finding that rates of DV-related calls for service after the first isolation period in early March 2020 either saw an initial increase (and then decreased or normalized), a sustained increase, or a decrease depending on the jurisdiction [22, 23]. The results here mirror and further expand these findings by demonstrating that rates can vary even within a region and in neighboring, demographically similar cities. Furthermore, we build on this literature by using statistical methods to determine inflection points, rather than imposing markers for when we anticipated changes to occur. Finally, we provide community context that might help explain rates of DV assault when mapped onto local public health mandates, which vary considerably state- and nationwide.

Although these data are informative and can give us a rough snapshot of what is happening in local communities, they should be interpreted with caution. Most importantly, because it is publicly available data provided at the discretion of participating police agencies, there is no way to verify accuracy beyond verification done by participating sites. Additionally, although the intention was to examine DV across Orange County to obtain an accurate and representative trajectory of DV pre- and post-lockdown, not all agencies provided DV data; thus, results only apply to their respective agencies and cities. Furthermore, although mostly uniform, there are discrepancies in how agencies report incidents, making it difficult to ascertain if the proportions across agencies can be compared when some agencies do not provide specific penal codes and instead classify events themselves before making their data available. Finally, it is possible that missing data from weeks in May and June, which correspond with the gradual loosening of restrictions after the first lockdown, contain important data that may indicate fluctuations within these months. Likewise, missing data from Santa Ana in late 2020 preclude us from detecting any changes in DV rates within this period. Still, results of available data suggest relative stability.

## Conclusion

Since the beginning of the pandemic, numerous advocates, service centers, and organizations foreshadowed an uptick in DV, given necessary stay-at-home directives and mandates that would isolate victims with their abusers. Shortly thereafter, sheriffs’ offices, shelters, agencies, hotlines, and journalists reported on this realized surge. It is worth noting once again that DV analyzed and reported herein are DV assaults as reported by police agencies. All other types of DV (e.g., psychological, emotional, stalking, sexual) are not reflected in these counts and are, in fact, rarely reported to police [24]. Moreover, even in an emergency, not all DV assault victims will make the call to the police or get the opportunity to do so. Thus, the sheer number of assault incidents reported here, while alarming, likely vastly underestimates the scope of domestic violence occurring in these communities. The methods presented in this paper could empower communities to track violent crimes like DV. While an imperfect data source, police records are publicly available, and the results of analyses can help serve as an immediate call to action.

## Data Availability

The data used and/or analyzed during the current study are available from the corresponding author on reasonable request.
